# O Escore Gensini e a Carga Trombótica Adicionam Valor Preditivo ao Escore SYNTAX na Detecção de No-Reflow após Infarto do Miocárdio

**DOI:** 10.36660/abc.20200045

**Published:** 2021-02-22

**Authors:** Luís Carlos V Matos, Luiz Sergio Carvalho, Rodrigo Modolo, Simone Santos, José Carlos Quinaglia e Silva, Osório Luis Rangel de Almeida, Andrei C. Sposito

**Affiliations:** 1 Escola Superior de Ciências da Saúde BrasíliaDF Brasil Escola Superior de Ciências da Saúde,Brasília, DF - Brasil; 2 Universidade Estadual de Campinas CampinasSP Brasil Universidade Estadual de Campinas,Campinas, SP - Brasil; 3 Hospital Brasília BrasíliaDF Brasil Hospital Brasília – Ecocardiografia,Brasília, DF - Brasil; 4 Eccos Diagnóstico Cardiovascular Avançado BrasíliaDF Brasil Eccos Diagnóstico Cardiovascular Avançado,Brasília, DF - Brasil; 5 Hospital de Base do Distrito Federal IGESDF BrasíliaDF Brasil Hospital de Base do Distrito Federal - IGESDF,Brasília, DF - Brasil

**Keywords:** Intervenção Coronária Percutânea/métodos, Infarto do Miocárdio, Aterosclerose, Trombose, Placa Aterosclerótica, Embolização Terapêutica

## Abstract

**Fundamento:**

O fenômeno de *no-reflow* após a intervenção coronária percutânea está associado a um pior prognóstico em pacientes com infarto do miocárdio com supradesnivelamento do segmento ST (IAMCSST). O escore SYNTAX é um bom preditor de *no-reflow*.

**Objetivo:**

Nosso objetivo foi avaliar se a carga aterosclerótica (escore Gensini) e a carga trombótica na artéria coronária culpada melhorariam a capacidade do escore SYNTAX para detectar o *no-reflow*.

**Métodos:**

Neste estudo coorte prospectivo, foram estudados pacientes com IAMCSST consecutivos que se apresentaram dentro de 12 horas a partir do início dos sintomas. O no-reflow foi definido como fluxo TIMI < 3 ou fluxo TIMI =3 mas grau de blush miocárdico (*myocardial blush grade*) < 2. A carga trombótica foi quantificada de acordo com o grau TIMI de trombo (0 a 5).

**Resultados:**

Foram incluídos 481 pacientes no estudo, com idade média de 61±11 anos. O fenômeno de no-reflow ocorreu em 32,8% dos pacientes. O escore SYNTAX (OR=1,05, IC95% 1,01–1,08, p<0,01), a carga trombótica (OR=1,17, IC95% 1,06–1,31, p<0,01), e o escore Gensini (OR=1,37, IC95% 1,13–1,65, p<0,01) foram preditores independentes do no-reflow. Os escores combinados apresentaram uma maior área sob a curva quando comparados ao escore SYNTAX isolado (0,78 [0,73–0,82] vs 0,73 [0,68–0,78], p=0,03). A análise da melhora da reclassificação líquida (NRI) categórica (0,11 [0,01–0,22], p=0,02) e contínua (NRI>0) (0,54 [0,035–0,73], p<0.001) mostrou melhora na capacidade preditiva do no-reflow no modelo combinado, com melhora da discriminação integrada (IDI) de 0,07 (0,04–0,09, p<0,001).

**Conclusões:**

Nossos achados sugerem que, em pacientes com IAMCSST submetidos à intervenção coronária percutânea, a carga aterosclerótica e a carga trombótica na artéria culpada adicionam valor preditivo ao escore SYNTAX na detecção do fenômeno no-reflow. (Arq Bras Cardiol. 2021; [online].ahead print, PP.0-0)

## Introdução

A intervenção coronária percutânea (ICP) é o método de reperfusão de escolha para infarto do miocárdio com supradesnivelamento do segmento ST (IAMCSST).^1^ Além de recuperar a patência do lúmen arterial, o objetivo dessa intervenção é promover o fluxo sanguíneo na microcirculação coronária.^2^ Contudo, um em cada três pacientes permanece com o fluxo microvascular reduzido apesar da restauração da patência da artéria coronária epicárdica. Esse fenômeno é conhecido como “no-reflow” (NR)^2,3^ O NR está associado a uma maior incidência de insuficiência cardíaca, choque cardiogênico, e morte.^3-5^

Um número considerável de marcadores de obstrução microvascular já foi descrito, particularmente idade e tempo de reperfusão.^6,7^ Mais recentemente, foi demonstrado que a complexidade anatômica para ICP, estimada pelo escore SYNTAX, também pode estar relacionada com um maior risco de NR.^8-10^ Uma vez que a ocorrência de NR é maior que a desses marcadores, é possível que existam outros marcadores de importância clínica. Neste contexto, é plausível a hipótese de que a carga aterosclerótica e trombótica adicione valor preditivo ao escore SYNTAX, idade, e tempo de reperfusão na predição de NR. O presente estudo teve como objetivo testar essa hipótese.

## Métodos

### Seleção da amostra

Este estudo baseou-se em uma subanálise do Brasilia Heart Study (BHS), cujo delineamento foi descrito previamente.^11^ Em resumo, o BHS é um estudo coorte, prospectivo, unicêntrico, de pacientes consecutivos com IAMCSST que se apresentaram nas primeiras 24 horas desde o início dos sintomas. IAMCSST foi definido segundo os critérios: 1) elevação do segmento ST em pelo menos 1 mm no plano frontal ou 2 mm no plano horizontal em duas derivações contíguas, ou novo bloqueio de ramo esquerdo pelo eletrocardiograma; 2) resultado positivo para o marcador de necrose miocárdica, definido como CK-MB>25 U/L, correspondendo a valores acima do percentil 99. Pacientes submetidos à ICP nas primeiras 12 horas do IAMCSST foram considerados elegíveis para o estudo. Os participantes assinaram um termo de consentimento e o estudo foi aprovado por um comitê de ética em pesquisa da instituição. Todos os procedimentos estavam de acordo com os padrões éticos do comitê institucional para pesquisa envolvendo humanos e com a declaração de Helsinki de 1964 e suas últimas revisões, ou padrões éticos comparáveis.

### Análise angiográfica

Todos os angiogramas foram revisados por dois cardiologistas intervencionistas experientes, que interpretaram as imagens de maneira independente, e avaliaram os seguintes parâmetros: fluxo coronário: fluxo TIMI;^12^ 2) perfusão miocárdica: *myocardial blush grade* (MBG);^13^ 3) carga trombótica: escala de trombos TIMI;^14^ 4) escore angiográfico SYNTAX,^8^ e o escore Gensini modificado.^15^ Os escores foram obtidos dos angiogramas de diagnóstico antes de qualquer intervenção. Os dois cardiologistas entraram em consenso nas interpretações dos achados, com variabilidade intraobservador e entre observador de 5%.

NR foi definido como um fluxo TIMI < 3 ou TIMI = 3 mas MBG<2 na angiografia coronária realizada após ICP da artéria relacionada ao IAMCSST.

### Análise estatística

Os dados quantitativos foram expressos em média e desvio padrão. Os grupos foram comparados pelo teste t de Student para variáveis contínuas paramétricas ou o teste de Mann-Whitney para variáveis contínuas não paramétricas, e pelo teste qui-quadrado para variáveis categóricas. Regressão logística binária foi usada para determinar preditores do fenômeno NR após reperfusão em modelo não ajustado (modelo 1) e ajustado (modelo 2) para escore GRACE e tempo de reperfusão (período entre início de sintomas e reperfusão) após reperfusão. Análise da curva característica de operação do receptor (curva ROC) foi realizada para determinar a capacidade preditiva dos modelos. A melhora da reclassificação líquida (NRI) e a melhora da discriminação integrada (IDI) foram usadas para determinar melhoras obtidas com a adição de novos preditores. Análise estatística foi realizada usando o programa SPSS para Mac, versão 23.0, e o programa R para Mac versão 3.4.2. Um valor de p < 0,05 foi considerado estatisticamente significativo.

## Resultados

Um total de 481 pacientes submetidos à ICP na fase aguda do IAMCSST foi incluído no estudo. A idade média dos pacientes foi 61 ± 11 anos, e 74,6% eram homens, 58,0% hipertensos, 54,2% sedentários, 38,0% fumantes, e 30,7% diabéticos. O fenômeno de NR ocorreu em 32,8% dos pacientes (n=158), os quais foram comparados com aqueles que apresentaram ótima reperfusão (n=323). Características clínicas e bioquímicas de ambos os grupos são descritas na Tabela 1.

**Tabela 1:** – Características clínicas e bioquímicas de 481 pacientes submetidos à intervenção coronária percutânea por infarto do miocárdio com supradesnivelamento do segmento ST que apresentaram reperfusão adequada ou ausência de reperfusão (fenômeno no-reflow) após o procedimento

Variáveis paramétricas	Total (n = 481)	Reperfusão adequada (n = 323)	No-reflow (n = 158)	Valor p^*^
**média ± DP**	**média ± DP**	**média ± DP**
Idade (anos)	61±11	61±11	61±12	0,64
IMC (kg.m-2)	27,0±4,2	26,8±3,9	27,3±4,7	0,32
GRACE na admissão hospitalar	136±26	135±27	137±24	0,46
Colesterol total no primeiro dia (mg.mL^-1^)	192±48	192±45	191±53	0,83
HDL-c no primeiro dia (mg.mL^-1^)	40±11	38±10	38±11	0,63
HbA1c (%)	6,5±1,8	6,5±1,8	6,4±1,7	0,64
**Variáveis não paramétricas**	**Mediana (Q1 - Q3)**	**Mediana (Q1 - Q3)**	**Mediana (Q1 - Q3)**	**Valor p^†^**
Tempo de reperfusão	111 (60 - 210)	96 (60 - 206)	120 (60 - 239)	0,42
LDL-c no primeiro dia (mg.mL^-1^)	117 (93 - 143)	117 (97 - 145)	118 (94 - 141)	0,50
Triglicerídeos no primeiro dia (mg.mL^-1^)	134 (87 - 207)	135 (90 - 215)	133 (84 - 195)	0,11
**Variáveis categóricas**	**ƒ (%)**	**ƒ (%)**	**ƒ (%)**	**Valor p^ǂ^**
Homens, n (%)	359 (75)	235 (73)	124 (78)	0,34
DM, n (%)	148 (31)	94 (29)	54 (34)	0,34
Hipertensão, n (%)	279 (58)	187 (58)	92 (58)	0,97
Acidente vascular cerebral, n (%)	21 (4)	11 (3)	10 (6)	0,24
Tabagismo, n (%)	183 (38)	127 (39)	56 (35)	0,35
Sedentarismo, n (%)	261 (54)	179 (55)	82 (52)	0,37
ICP previa, n (%)	26 (5)	15 (5)	11 (7)	0,35
CABG, n (%)	4 (0,8)	3 (0,9)	1 (0,6)	0,86
Killip >1, n (%)	52 (11)	31 (10)	21 (13,2)	0,44

DP: desvio padrão; Q1: primeiro quartil; Q3: terceiro quartil. CABG: coronary artery bypass grafting; GRACE: Global Registry of Acute Coronary Eventos; DM: diabetes mellitus tipo 2; ICP: intervenção coronária percutânea; IMC: índice de massa corporal; HbA1C, hemoglobina glicada; LDL, lipoproteína de baixa densidade; HDL, lipoproteína de alta densidade. * teste t de Student não pareado; † teste Mann-Whitney não paramétrico; ǂ teste qui-quadrado.

**Figura 1:**
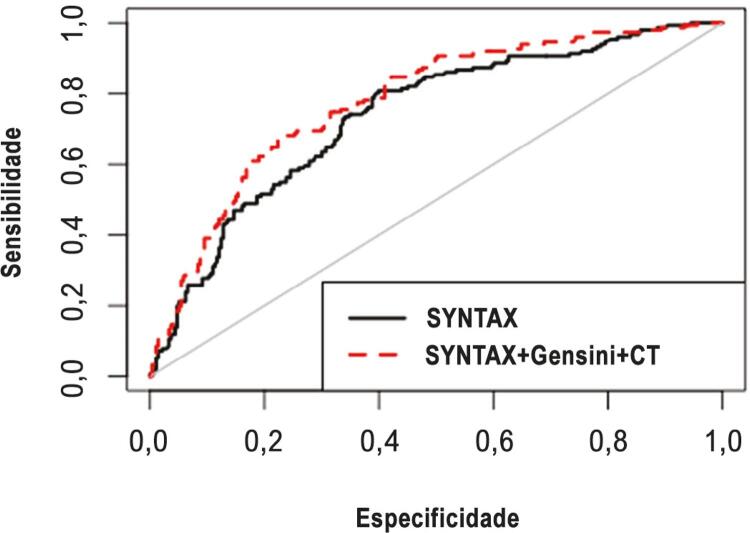
Comparação das curvas ROC entre escores SYNTAX, Gensini, e de carga trombótica (CT) combinados e o escore SYNTAX isolado.

O escore Gensini, o escore Gensini da artéria culpada, o escore SYNTAX, e a carga trombótica foram significativamente maiores no grupo NR que no grupo com reperfusão adequada (Tabela 2). Ambos os modelos de regressão logística (ajustado e não ajustado) mostraram que o escore SYNTAX, o escore Gensini, e a carga trombótica foram preditores independentes do NR (Tabela 3). A análise da curva ROC mostrou que os modelos com escores combinados apresentaram maior área sob a curva ROC que o modelo que apresentava somente o escore SYNTAX [0,778 (0,733 – 0,823) vs. 0.737 (0,688 – 0,786)] (Figura 1).

**Tabela 2:** – Parâmetros angiográficos de 481 pacientes submetidos à intervenção coronária percutânea por infarto do miocárdio com supradesnivelamento do segmento ST

Parâmetros	n = 481	Reperfusão adequada n = 323	No-reflow n = 158	p
Escore Gensini	100±70	82±62	139±69	<0,001
Escore Gensini – artéria culpada	62±49	48±38	87±56	<0,001
Escore SYNTAX	12±10	9±8	17±10	<0,001
Carga trombótica – artéria culpada, n (%)	181 (37,6)	89 (27,5)	92 (58,2)	<0,001
Escala TIMI de trombo, n (%)				<0,001
0	300 (63)	234 (72)	66 (42)	
1	9 (1,9)	8 (2,5)	1 (0,6)	
2	21 (4,4)	13 (4)	8 (5)	
3	18 (3,7)	14 (4,3)	4 (2,5)	
4	18 (3,7)	11 (3,4)	7 (4,4)	
5	115 (24)	43 (13,3)	72 (46)	

*Valores expressos em média ± desvio padrão; TIMI: thrombolysis in myocardial infarction.*

**Tabela 3:** – Modelo de regressão logística dos escores SYNTAX, Gensini, e carga trombótica como preditores do fenômeno de no-reflow

Modelos	OR (IC95%)	p
**Modelo 1 (não ajustado)**		
Idade	1,02 (1,00-1,03	0,037
Tempo de reperfusão	1,00 (1,00-1,01)	0,154
Escore SYNTAX	1,10 (1,07-1,12)	<0,001
Carga trombótica	1,38 (1,26-1,51)	<0,001
Escore Gensini	1,76 (1,50-2,07)	<0,001
Modelo 2 (mutivariado)*		
Idade^†^	1,01 (0,98-1,02)	0,325
Tempo de reperfusão	0,98 (0,99-1,00)	0,645
Escore SYNTAX	1,05 (1,01-1,08)	<0,01
Carga trombótica	1,17 (1,06-1,31)	<0,01
Escore Gensini	1,37 (1,13-1,65)	<0,01

IC: intervalo de confiança; OR: odds ratio. Todos os modelos foram ajustados para o escore GRACE e tempo de reperfusão (tempo entre início de sintomas e reperfusão). * ajustado para escore GRACE score; † não inclui o escore GRACE

A NRI e a estratificação do NR entre o escore SYNTAX isolado e os escores combinados (SYNTAX, Gensini e carga trombótica) são apresentadas na Tabela 4. As análises da NRI contínua e categórica mostraram melhora na capacidade preditiva do NR no modelo combinado, o que também foi indicado pela melhora discriminada integrada (IDI) (Tabela 5).

**Tabela 4:** – Melhora da reclassificação líquida e estratificação do fenômeno no-reflow entre os escores SYNTAX, Gensini, e de carga trombótica combinados e o escore SYNTAX isolado

Escore SYNTAX isolado	Escores combinados			
	Baixo	Intermediário	Alto	Reclassificação
Baixo	301	26	1	8%
Intermediário	23	22	34	72%
Alto	2	9	6	65%

**Tabela 5:** – Melhora da reclassificação líquida (NRI) contínua e categórica, melhora da discriminação integrada (IDI) e valor preditivo do no-reflow entre os escores SYNTAX, Gensini, e de carga trombótica combinados e o escore SYNTAX isolado

Variáveis	NRI	IC95%	p
Contínuas	0,54	0,351-0,7347	<0,001
Categóricas	0,12	0,0119-0,2223	<0,02
IDI	0,066	0,040-0,092	<0,001

*IC: intervalo de confiança.*

## Discussão

Entre os principais achados deste estudo, encontramos que (1) o escore SYNTAX, o escore Gensini, e a carga trombótica foram preditores independentes do NR; e (2) a combinação dos escores de carga aterosclerótica e de carga trombótica com o escore SYNTAX aumentou o valor preditivo do escore SYNTAX na detecção do fenômeno NR.

Apesar de o escore SYNTAX ser um bom preditor de disfunção microvascular, a carga aterosclerótica não é considerada no algoritmo, uma vez que ele exclui lesões obstrutivas com estenoses menores que 50%. Ainda, a carga trombótica também não é considerada no algoritmo SYNTAX, uma vez que ele atribui um escore relativamente baixo para a presença ou ausência de trombo.^8^ Contudo, o escore Gensini é bastante representativo da carga aterosclerótica, uma vez que pontua lesões com pelo menos 25% de estenose^15,16^ e se associa de maneira significativa com a carga de placa e a área da placa medidas por ultrassom intracoronário.^17^ Valores altos no escore Gensini podem indicar doença de múltiplos vasos e um aumento na resistência microvascular, ambos associados com NR.^5,10,18^

No presente estudo, o escore Gensini foi um preditor independente de NR. Modolo et al.,^19^ mostraram que o escore Gensini total e o escore Gensini da artéria culpada foram mais altos nos indivíduos com NR que nos indivíduos com reperfusão adequada^19^ No entanto, a gravidade da estenose do lúmen não é o único fator angiográfico preditor de disfunção microvascular. De fato, alterações morfológicas da placa, tais como o alto teor lipídico, centro necrótico extenso, e grande quantidade de placa atenuada também são fortes preditores de NR,^20,21^ sugerindo que alterações no volume e no conteúdo da placa causem autorregulação inadequada e liberação local de vasoconstritores, aumentando a formação de trombos, microembolização de leitos arteriais e obstrução microvascular.

No presente estudo, 58,5% dos pacientes no grupo NR apresentaram trombos na artéria culpada; 50,4% desses com alta carga trombótica (graus 4 e 5 da escala TIMI de trombos), uma possível razão para a associação com o NR. Em uma grande coorte de pacientes com IAMCSST submetidos à ICP, uma grande carga trombótica foi associada à NR (4,0 vs 0,5, p<0,001) e à embolização distal (17,3 vs 3,4, p<0,001).^22^

O escore SYNTAX foi significativamente mais alto no grupo com NR que no grupo com reperfusão adequada (17±10 vs 9±8), e um preditor independente de NR. Em um estudo prévio, o escore SYNTAX foi um preditor de NR, e um escore SYNTAX >21 duplicou o risco de se desenvolver NR.^9^ A oclusão total da artéria relacionada ao IAMCSST, local da oclusão (artéria coronária principal esquerda ou artéria descendente anterior esquerda), presença de trombos, lesões mais extensas, lesões na bifurcação e doença de múltiplos vasos são fatores associados com escores SYNTAX aumentados e podem explicar a associação com NR.^9,10,23^

No presente estudo, o fenômeno NR ocorreu em 32,8% dos casos, fluxo TIMI <3 ou fluxo TIMI =3 e MBG <2 como critério angiográfico. A incidência de NR é muito maior no IAMCSST que em ICP eletivo, sendo relatada em 30 a 50% dos pacientes submetidos à ICP primária por IAMCSST.^3^ Rezkalla et al.,^3^ investigando NR em pacientes com IAMCSST, encontraram uma prevalência de 32% quando avaliados por TIMI e de 52% quando avaliados por MBG.

Idade é um importante marcador de NR. Pacientes idosos têm maior carga de placas, aterosclerose coronária difusa e calcificação vascular grave, o que pode contribuir para a disfunção microvascular.^24,25^ Zhou et al.,^6^ identificaram que idade >65 anos (OR= 1,470, IC95%1,460-1,490, p=0,007) foi um preditor independente de NR.^6^Em nosso estudo, idade foi um fator preditor de NR na análise univariada, mas essa relação não se manteve na análise multivariada.

A reperfusão tardia está associada com NR. Estudos prévios mostraram que pacientes com tempo de reperfusão mais longos (>6 horas) apresentaram um aumento significativo no NR.^6,7^ No entanto, um estudo utilizando um ponto de corte mais baixo (<6 horas) do início dos sintomas não indicou que a apresentação tardia fosse um preditor independente de NR.^23^ Em nosso estudo, o tempo de reperfusão foi 2,94 horas no grupo com NR e de 2,5 horas no grupo com reperfusão adequada. A análise multivariada ajustada para o escore GRACE não indicou o tempo de reperfusão como um preditor de NR.

A fisiopatologia do fenômeno de NR é multifatorial e envolve suscetibilidade individual, lesão relacionada à isquemia, lesão relacionada à reperfusão, e embolização distal.^26^ Durante a ICP, no IAMCSST, a embolização distal do trombo e os componentes da placa aterosclerótica são mecanismos importantes envolvidos na patogênese do NR.^27,28^ O material aterotrombótico liberado causa obstrução mecânica, vasoconstrição pela liberação de serotonina, tromboxano A2, e endotelina, e disfunção endotelial pela expressão aumentada de fator de necrose tumoral alfa (TNFα).^28-30^ Ainda, a liberação de micropartículas plaquetárias e endoteliais está associada à perfusão miocárdica reduzida avaliada por MBG e à maior carga trombótica.^31^ Os neutrófilos liberam microfilamentos , os Neutrophil Extracellular Traps (NETs) que promovem trombose e inflamação na artéria culpada, contribuindo para morte dos miócitos.^32,33^

Uma limitação deste estudo é seu caráter unicêntrico. Além disso, a angiografia coronária tem capacidade limitada para estimar tanto a carga trombótica como a carga aterosclerótica quando comparada à ultrassonografia intracoronária e à tomografia de coerência ótica.

## Conclusão

A carga aterosclerótica avaliada pelo escore Gensini e a carga trombótica na artéria culpada adicionam valor preditivo ao escore SYNTAX na detecção de NR após ICP em pacientes com IAMCSST.

## References

[1] 1. Ibanez B, James S, Agewall S, Antunes MJ, Bucciarelli-Ducci C, Bueno H, et al. 2017 ESC Guidelines for the management of acute myocardial infarction in patients presenting with ST-segment elevation: The Task Force for the management of acute myocardial infarction in patients presenting with ST-segment elevation of the European Society of Cardiology (ESC). Eur Heart J. 2018;39(2):119-77. 10.1093/eurheartj/ehx393 28886621

[2] 2. Heusch G, Gersh BJ. The pathophysiology of acute myocardial infarction and strategies of protection beyond reperfusion: A continual challenge. Eur Heart J. 2017;38(11):774-84. 10.1093/eurheartj/ehw224 27354052

[3] 3. Rezkalla SH, Dharmashankar KC, Abdalrahman IB, Kloner RA. No-reflow phenomenon following percutaneous coronary intervention for acute myocardial infarction: Incidence, outcome, and effect of pharmacologic therapy. J Interv Cardiol. 2010;23(5):429-36. 10.1111/j.1540-8183.2010.00561.x 20819117

[4] 4. De Waha S, Patel MR, Granger CB, Ohman EM, Maehara A, Eitel I, et al. Relationship between microvascular obstruction and adverse events following primary percutaneous coronary intervention for ST-segment elevation myocardial infarction: An individual patient data pooled analysis from seven randomized trials. Eur Heart J. 2017;38(47):3502-10. 10.1093/eurheartj/ehx414 29020248

[5] 5. Ndrepepa G, Tiroch K, Fusaro M, Keta D, Seyfarth M, Byrne RA, et al. 5-Year prognostic value of no-reflow phenomenon after percutaneous coronary intervention in patients with acute myocardial infarction. J Am Coll Cardiol. 2010;55(21):2383-89. 10.1016/j.jacc.2009.12.054 20488311

[6] 6. Zhou H, He X, Zhuang S, Wang J, Lai Y, Qi W, et al. Evaluation of clinical and procedural predictors of the no-reflow phenomenon in patients with acute myocardial infarction after primary percutaneous coronary intervention. Chinese J Emerg Med. 2013;22:280-6. 10.5847/wjem.j.issn.1920-8642.2014.02.003 PMC4129879 25215156

[7] 7. Mazhar J, Mashicharan M, Farshid A. Predictors and outcome of no-reflow post primary percutaneous coronary intervention for ST elevation myocardial infarction. Int J Cardiol Heart Vasc. 2016;10:8-12. 10.1016/j.ijcha.2015.11.002 PMC5441318 28616509

[8] 8. Sianos G, Morel MA, Kappetein AP, Morice MC, Colombo A, Dawkins K, et al. The SYNTAX Score: an angiographic tool grading the complexity of coronary artery disease. EuroIntervention. 2005;1(2):219-27. 19758907

[9] 9. Magro M, Nauta ST, Simsek C, Boersma E, van der Heide E, Regar E, et al. Usefulness of the SYNTAX Score to predict “no reflow” in patients treated with primary percutaneous coronary intervention for st-segment elevation myocardial infarction. Am J Cardiol. 2012;109(5):601-6. 10.1016/j.amjcard.2011.10.013 22177003

[10] 10. Sahin DY, Gür M, Elbasan Z, Kuloğlu O, Seker T, Kivrak A, et al. SYNTAX score is a predictor of angiographic no-reflow in patients with st elevation myocardial infarction treated with primary percutaneous coronary intervention. Coron Artery Dis. 2013;24(2):148-53. 10.1097/MCA.0b013e32835c4719 23363986

[11] 11. Sposito AC, Carvalho LS, Cintra RM, Araújo AL, Ono AH, Andrade JM, et al. Rebound inflammatory response during the acute phase of myocardial infarction after simvastatin withdrawal. Atherosclerosis. 2009;207(1):191-4. 10.1016/j.atherosclerosis.2009.04.008 19464010

[12] 12. TIMI Study Group. The Thrombolysis in Myocardial Infarction (TIMI) trial. Phase I findings. N Engl J Med. 1985;312(14):932-6. 10.1056/NEJM198504043121437 4038784

[13] 13. Van’t Hof A, Liem A, Suryapranata H, Hoorntje JC, de Boer MJ, Zijlstra F. Angiographic assessment of myocardial reperfusion in patients treated with primary angioplasty for acute myocardial infarction: myocardial blush grade. Zwolle Myocardial Infarction Study Group. Circulation. 1998;97(23):2302-6. 10.1161/01.cir.97.23.2302 9639373

[14] 14. Gibson C, de Lemos JA, Murphy SA, Marble SJ, McCabe CH, Cannon CP, et al. Combination therapy with abciximab reduces angiographically evident thrombus in acute myocardial infarction a TIMI 14 substudy. Circulation. 2001;103(21):2550-4. 10.1161/01.cir.103.21.2550 11382722

[15] 15. Ringqvist I, Fisher LD, Mock M, Davis KB, Wedel H, Chaitman BR, et al. Prognostic value of angiographic indices of coronary artery disease from the Coronary Artery Surgery Study (CASS). J Clin Invest. 1983;71(6):1854-66. 10.1172/JCI110941 PMC370391 6863543

[16] 16. Gensini GG. A more meaningful scoring system for determining the severity of coronary heart disease. Am J Cardiol. 1983;51(3):606. 10.1016/s0002-9149(83)80105-2 6823874

[17] 17. Neeland IJ, Patel RS, Eshtehardi P, Dhawan S, McDaniel MC, Rab ST, et al. Coronary angiographic scoring systems: An evaluation of their equivalence and validity. Am Heart J. 2012;164(4):547-52. 10.1016/j.ahj.2012.07.007 PMC3913177 23067913

[18] 18. Melikian N, Vercauteren S, Fearon WF, Cuisset T, MacCarthy PA, Davidavicius G, et al. Quantitative assessment of coronary microvascular function in patients with and without epicardial atherosclerosis. EuroIntervention. 2010;5(8):939-45. 20542779

[19] 19. Modolo R, Figueiredo VN, Moura FA, Almeida B, Quinaglia e Silva JC, Nadruz W Jr, et al. Coronary artery calcification score is an independent predictor of the no-reflow phenomenon after reperfusion therapy in acute myocardial infarction. Coron Artery Dis. 2015;26(7):562-6. 10.1097/MCA.0000000000000269 26010531

[20] 20. Tanaka A, Kawarabayashi T, Nishibori Y, Sano T, Nishida Y, Fukuda D, et al. No-reflow phenomenon and lesion morphology in patients with acute myocardial infarction. Circulation. 2002;105(18):2148-52. 10.1161/01.cir.0000015697.59592.07 11994247

[21] 21. Wu X, Mintz GS, Xu K, Lansky AJ, Witzenbichler B, Guagliumi G, et al. The relationship between attenuated plaque identified by intravascular ultrasound and no-reflow after stenting in acute myocardial infarction: The HORIZONS-AMI (Harmonizing Outcomes with Revascularization and Stents in Acute Myocardial Infarction) trial. JACC Cardiovasc Interv. 2011;4(5):495-02. 10.1016/j.jcin.2010.12.012 21596321

[22] 22. Sianos G, Papafaklis MI, Daemen J, Vaina S, van Mieghem CA, van Domburg RT, et al. Angiographic stent thrombosis after routine use of drug-eluting stents in ST-segment elevation myocardial infarction: the importance of thrombus burden. J Am Coll Cardiol. 2007;50(7):573-83. 10.1016/j.jacc.2007.04.059 17692740

[23] 23. Chan W, Stub D, Clark DJ, Ajani AE, Andrianopoulos N, Brennan AL, et al. Usefulness of transient and persistent no reflow to predict adverse clinical outcomes following percutaneous coronary intervention. Am J Cardiol. 2012;109(4):478-85. 10.1016/j.amjcard.2011.09.037 22176999

[24] 24. Kirma C, Izgi A, Dundar C, Tanalp AC, Oduncu V, Aung SM, et al. Clinical and procedural predictors of no-reflow phenomenon after primary percutaneous coronary interventions: experience at a single center. Circ J. 2008;72(5):716-21. 10.1253/circj.72.716 18441449

[25] 25. Ruiz-García J, Lerman A, Weisz G, Maehara A, Mintz GS, Fahy M, et al. Age- and gender-related changes in plaque composition in patients with acute coronary syndrome: the PROSPECT study. EuroIntervention. 2012;8(8):929-38. 10.4244/EIJV8I8A142 23253546

[26] 26. Niccoli G, Scalone G, Lerman A, Crea F. Coronary microvascular obstruction in acute myocardial infarction. Eur Heart J. 2016;37(13):1024-33. 10.1093/eurheartj/ehv484 26364289

[27] 27. Henriques JP, Zijlstra F, Ottervanger JP, de Boer MJ, van’t Hof AW, Hoorntje JC, et al. Incidence and clinical significance of distal embolization during primary angioplasty for acute myocardial infarction. Eur Heart J. 2002;23(14):1112-7. 10.1053/euhj.2001.3035 12090749

[28] 28. Shome JS, Perera D, Plein S, Chiribiri A. Current perspectives in coronary microvascular dysfunction. Microcirculation. 2017;24(1):1-13. 10.1111/micc.12340 27976459

[29] 29. Heusch G, Skyschally A, Kleinbongard P. Coronary microembolization and microvascular dysfunction. Int J Cardiol. 2018;258:17-23. 10.1016/j.ijcard.2018.02.010 29429637

[30] 30. Heusch G, Kleinbongard P, Böse D, Levkau B, Haude M, Schulz R, et al. Coronary microembolization: From bedside to bench and back to bedside. Circulation. 2009;120(18):1822-36. 10.1161/CIRCULATIONAHA.109.888784 19884481

[31] 31. Porto I, Biasucci LM, De Maria GL, Leone AM, Niccoli G, Burzotta F, et al. Intracoronary microparticles and microvascular obstruction in patients with ST elevation myocardial infarction undergoing primary percutaneous intervention. Eur Heart J. 2012;33(23):2928-38. 10.1093/eurheartj/ehs065 22453653

[32] 32. Mangold A, Alias S, Scherz T, Hofbauer T, Jakowitsch J, Panzenböck A, et al. Coronary neutrophil extracellular trap burden and deoxyribonuclease activity in ST-elevation acute coronary syndrome are predictors of ST-segment resolution and infarct size. Circ Res. 2015:116(7):1182-92. 10.1161/CIRCRESAHA.116.304944 25547404

[33] 33. Stakos DA, Kambas K, Konstantinidis T, Mitroulis I, Apostolidou E, Arelaki S, et al. Expression of functional tissue factor by neutrophil extracellular traps in culprit artery of acute myocardial infarction. Eur Heart J. 2015;36(22):1405-14. 10.1093/eurheartj/ehv007 PMC4458286 25660055

