# Supplementary material for: O Escore Gensini e a Carga Trombótica Adicionam Valor Preditivo ao Escore SYNTAX na Detecção de No-Reflow após Infarto do Miocárdio
Source: Arq Bras Cardiol. 2021 Feb 22;116(3):466–72. [Article in Portuguese] doi: 10.36660/abc.20200045 (PMC8159555; doi:10.36660/abc.20200045)
Supplement: Translation

# Translation: Gensini Score and Thrombus Burden Add Predictive Value to the SYNTAX Score in Detecting No-Reflow after Myocardial Infarction

**Authors:** Luís Carlos V Matos, Luiz Sergio Carvalho, Rodrigo Modolo, Simone Santos, José Carlos Quinaglia e Silva, Osório Luis Rangel de Almeida, Andrei C. Sposito

**Affiliations:** 1 Brazil Escola Superior de Ciências da Saúde, Brasília, DF - Brazil; 2 Brazil Universidade Estadual de Campinas, Campinas, SP - Brazil; 3 Brazil Hospital Brasília – Ecocardiografia, Brasília, DF - Brazil; 4 Brazil Eccos Diagnóstico Cardiovascular Avançado, Brasília, DF - Brazil; 5 Brazil Hospital de Base do Distrito Federal - IGESDF, Brasília, DF - Brazil

**Keywords:** Percutaneous Coronary Intervention/methods, Myocardial Infarction, Atheroscclerosis, Thrombosis, Plaque, Atherosclerotic, Embolization, Therapeutic

## Abstract

**Background:**

No-reflow after percutaneous coronary intervention is associated with poor prognosis in patients with ST-segment elevation myocardial infarction (STEMI). SYNTAX score is a good predictor of no-reflow.

**Objective:**

We aimed to evaluate whether atherosclerotic burden (Gensini score) and thrombus burden in the culprit coronary artery would improve the ability of the SYNTAX score to detect no-reflow.

**Methods:**

In this prospective cohort study, consecutive patients with STEMI who presented within 12 h of onset of symptoms were selected for this study. No-reflow was defined as TIMI flow < 3 o r TIMI flow = 3 but myocardial blush grade <2. Thrombus burden was quantified according to the TIMI thrombus grade scale (0 to 5).

**Results:**

A total of 481 patients were included (mean age 61±11 years). No-reflow occurred in 32.8%. SYNTAX score (OR=1.05, 95%CI 1.01–1.08, p<0.01), thrombus burden (OR=1.17, 95%CI 1.06–1.31, p<0.01), and Gensini score (OR=1.37, 95%CI 1.13–1.65, p<0.01) were independent predictors of no-reflow. Combined scores had a larger area under the curve than the SYNTAX score alone (0.78 [0.73–0.82] vs 0.73 [0.68–0.78], p=0.03). Analyses of both categorical (0.11 [0.01–0.22], p=0.02), and continuous net reclassification improvement (NRI>0) (0.54 [0.035–0.73], p<0.001) showed improvement in the predictive ability of no-reflow in the combined model, with integrated discrimination improvement (IDI) of 0.07 (0.04–0.09, p<0.001).

**Conclusions:**

Our findings suggest that, in patients with STEMI undergoing percutaneous coronary intervention, atherosclerotic burden and thrombus burden in the culprit artery add predictive value to the SYNTAX score in detecting the no-reflow phenomenon. (Arq Bras Cardiol. 2021; [online].ahead print, PP.0-0)

## Introduction

Percutaneous coronary intervention (PCI) is the reperfusion strategy of choice for ST-segment elevation myocardial infarction (STEMI).^1^ More than to restore the patency of the arterial lumen, the objective of this intervention is to provide blood flow in the coronary microcirculation.^2^ However, in one out of three patients, the microvascular flow remains diminished despite restoration of epicardial coronary artery patency, a phenomenon named no-reflow (NR).^2,3^ The NR is associated with an increased incidence of heart failure, cardiogenic shock, and death.^3-5^

A sizeable number of markers for microvascular obstruction have been described, particularly age and time to reperfusion.^6,7^ More recently, it has been shown that anatomic complexity for PCI, as estimated by the SYNTAX score, may also relate to a higher risk for NR.^8-10^ As NR occurs more frequently than the above-mentioned markers, it is possible that other clinically relevant markers exist. In this context, it is hypothetically plausible that atherosclerotic and thrombotic burden may add predictive value to the SYNTAX score, age, and time to reperfusion in the prediction of NR. The present study was therefore designed to test this hypothesis.

## Methods

### Sample selection

This study was based on a subanalysis of the Brasilia Heart Study (BHS), whose design is described elsewhere.^11^ Briefly, the BHS is a single-center, prospective cohort study of consecutive patients with STEMI who presented within 24 h of onset of symptoms. STEMI was defined as follows: 1) ST-segment elevation of at least 1 mm in the frontal plane or 2 mm in the horizontal plane in two contiguous leads, or new left bundle branch block on electrocardiogram; 2) positive myocardial necrosis marker, defined as troponin I >0.04 ng/mL and CK-MB >25 U/L, corresponding to values above the 99th percentile. Patients undergoing PCI within 12 h of STEMI were eligible for the present study. Written informed consent was obtained from all participants, and the study was approved by the research ethics committee of the institution. All procedures were in accordance with the ethical standards of the institutional committee on human experimentation and with the 1964 Helsinki declaration and its later amendments or comparable ethical standards.

### Angiographic analysis

All angiograms were reviewed by two experienced interventional cardiologists who independently interpreted the images and evaluated the following parameters: 1) coronary flow: TIMI flow grade;^12^ 2) myocardial perfusion: myocardial blush grade (MBG);^13^ 3) thrombus burden: TIMI thrombus grade scale;^14^ 4) angiographic SYNTAX score^8^ and modified Gensini score.^15^.The scores were obtained from the diagnostic angiogram before any intervention. The two cardiologists agreed on the interpretation of findings, with an intraobserver and interobserver variability of 5%.

NR was defined as a TIMI flow grade < 3 o r T I M I f l o w g r a d e = 3 but MBG <2 at coronary angiography performed after PCI of the STEMI-related artery.

### Statistical analysis

Quantitative data were expressed as mean and standard deviation (SD). Groups were compared using Student’s *t* test for parametric continuous variables or the Mann-Whitney test for nonparametric continuous variables, and the chi-square test was used for categorical variables. Binary logistic regression was used to determine predictors of the NR phenomenon in models unadjusted (model 1) and adjusted (model 2) for GRACE score and reperfusion time (time between symptom onset and reperfusion) after reperfusion. Receiver operating characteristic (ROC) curve analysis was performed to determine the predictive ability of the models. Net reclassification improvement (NRI) and integrated discrimination improvement (IDI) were used to determine improvements with the addition of new predictors. Statistical analysis was performed using SPSS for Mac version 23.0 (SPSS Inc., Chicago, IL, USA), and R for Mac version 3.4.2. A p-value <0.05 was considered statistically significant.

## Results

A total of 481 patients undergoing PCI in the acute phase of STEMI were included in the present study. Mean patient age was 61 (SD 11) years, and 74.6% were men, 58.0% had hypertension, 54.2% were physically inactive, 38.0% were smokers, and 30.7% had diabetes. NR occurred in 32.8% of the patients (n=158), who were then compared to those who had optimal reperfusion (n=323). Clinical and biochemical characteristics of both groups are described in Table 1.

**Table 1:** – Clinical and biochemical characteristics of 481 patients undergoing percutaneous coronary intervention for ST-segment elevation myocardial infarction (STEMI) who showed optimal reperfusion or no-reflow after the procedure

Parametric variables	Total (n = 481)	Optimal reperfusion (n = 323)	No-reflow (n = 158)	P- value^*^
**mean ± SD**	**mean ± SD**	**mean ± SD**
Age (years)	61±11	61±11	61±12	0.64
BMI (kg.m-2)	27.0±4.2	26.8±3.9	27.3±4.7	0.32
GRACE on hospital admission	136±26	135±27	137±24	0.46
First-day total cholesterol (mg.mL-1)	192±48	192±45	191±53	0.83
First-day HDL-c (mg.mL-1)	40±11	38±10	38±11	0.63
HbA1c (%)	6.5±1.8	6.5±1.8	6.4±1.7	0.64
**Non-parametric variables**	**Median (Q1 - Q3)**	**Median (Q1 - Q3)**	**Median (Q1 - Q3)**	**P- value^†^**
Reperfusion time	111 (60 - 210)	96 (60 - 206)	120 (60 - 239)	0.42
First-day LDL-c (mg.mL-1)	117 (93 - 143)	117 (97 - 145)	118 (94 - 141)	0.50
First-day triglycerides (mg.mL-1)	134 (87 - 207)	135 (90 - 215)	133 (84 - 195)	0.11
**Categorical variables**	**ƒ (%)**	**ƒ (%)**	**ƒ (%)**	**P- value^**ǂ**^**
Male, n (%)	359 (75)	235 (73)	124 (78)	0.34
DM, n (%)	148 (31)	94 (29)	54 (34)	0.34
Hypertension, n (%)	279 (58)	187 (58)	92 (58)	0.97
Stroke, n (%)	21 (4)	11 (3)	10 (6)	0.24
Smoking, n (%)	183 (38)	127 (39)	56 (35)	0.35
Physical inactivity, n (%)	261 (54)	179 (55)	82 (52)	0.37
Previous PCI, n (%)	26 (5)	15 (5)	11 (7)	0.35
CABG, n (%)	4 (0.8)	3 (0.9)	1 (0.6)	0.86
Killip >1, n (%)	52 (11)	31 (10)	21 (13.2)	0.44

SD: standard deviation; Q1: first quartile; Q3: third quartile. CABG: coronary artery bypass grafting; GRACE: Global Registry of Acute Coronary Events; DM: type 2 diabetes mellitus; PCI: percutaneous coronary intervention; BMI: body mass index; HbA1C: glycosylated hemoglobin; LDL: low-density lipoprotein; HDL: high-density lipoprotein. * unpaired Student’s t test; † Non-parametric Mann-Whitney test; ǂ Chi-squared test.

Gensini score, Gensini score of the culprit artery, SYNTAX score, and thrombus burden were significantly higher in the NR group than in the optimal reperfusion group (Table 2). Both unadjusted and adjusted logistic regression models showed that SYNTAX score, Gensini score, and thrombus burden were independent predictors of NR (Table 3). ROC curve analysis showed that the model with combined scores had a larger area under the ROC curve than the model with the SYNTAX score alone (0.778 [0.733 - 0.823] vs. 0.737 [0.688 - 0.786]) (Figure 1).

**Table 2:** – Angiographic parameters of 481 patients undergoing percutaneous coronary intervention for ST-segment elevation myocardial infarction (STEMI)

Parameters	n = 481	Optimal reperfusion n = 323	No-reflow n = 158	p
Gensini score	100±70	82±62	139±69	<0.001
Gensini score – culprit artery	62±49	48±38	87±56	<0.001
SYNTAX score	12±10	9±8	17±10	<0.001
Thrombus burden – culprit artery, n (%)	181 (37.6)	89 (27.5)	92 (58.2)	<0.001
TIMI thrombus grade scale, n (%)				<0.001
0	300 (63)	234 (72)	66 (42)	
1	9 (1.9)	8 (2.5)	1 (0.6)	
2	21 (4.4)	13 (4)	8 (5)	
3	18 (3.7)	14 (4.3)	4 (2.5)	
4	18 (3.7)	11 (3.4)	7 (4.4)	
5	115 (24)	43 (13.3)	72 (46)	

*Values are expressed as mean ± standard deviation. TIMI: thrombolysis in myocardial infarction.*

**Table 3:** – Logistic regression model of the SYNTAX, Gensini, and thrombus burden scores as predictors of the no-reflow phenomenon

Models	OR (95%CI)	p
**Model 1 (unadjusted)**		
Age	1.02 (1.00-1.03	0.037
Reperfusion time	1.00 (1.00-1.01)	0.154
SYNTAX score	1.10 (1.07-1.12)	<0.001
Thrombus burden	1.38 (1.26-1.51)	<0.001
Gensini score	1.76 (1.50-2.07)	<0.001
Model 2 (multivariate)*		
Age^†^	1.01 (0.98-1.02)	0.325
Reperfusion time	0.98 (0.99-1.00)	0.645
SYNTAX score	1.05 (1.01-1.08)	<0.01
Thrombus burden	1.17 (1.06-1.31)	<0.01
Gensini score	1.37 (1.13-1.65)	<0.01

CI: confidence interval; OR: odds ratio. All models were adjusted for GRACE score and reperfusion time (time between symptom onset and reperfusion). * adjusted for GRACE score; ^†^ not including GRACE score.

**Figure 1:** Comparison of ROC curves between combined SYNTAX, Gensini, and thrombus burden (TB) scores and the SYNTAX score alone.

NRI and stratification of NR between the SYNTAX score alone and combined SYNTAX, Gensini, and thrombus burden scores are shown in Table 4. Both categorical and continuous NRI analyses showed improvement in the predictive ability of NR in the combined model, which was also indicated by the integrated discrimination improvement (IDI) (Table 5).

**Table 4:** – Net reclassification improvement (NRI) and stratification of no-reflow between combined SYNTAX, Gensini, and thrombus burden scores and the SYNTAX score alone

SYNTAX score alone	Combined scores			
	Low	Intermediate	High	Reclassification
Low	301	26	1	8%
Intermediate	23	22	34	72%
High	2	9	6	65%

**Table 5:** – Continuous and categorical net reclassification improvement (NRI), integrated discrimination improvement (IDI), and predictive value of no-reflow between combined SYNTAX, Gensini, and thrombus burden scores and the SYNTAX score alone

Variables	NRI	95%CI	p
Continuous	0.54	0.351-0.7347	<0.001
Categorical	0.12	0.0119-0.2223	<0.02
IDI	0.066	0.040-0.092	<0.001

*CI: confidence interval.*

## Discussion

Among the main findings of this study, we found that (1) SYNTAX score, Gensini score, and thrombus burden were all independent predictors of NR; and (2) the combination of atherosclerotic burden and thrombus burden scores with the SYNTAX score increased the predictive value of the SYNTAX score in detecting the NR phenomenon.

Although the SYNTAX score is a good predictor of microvascular dysfunction, total atherosclerotic burden is not considered in the algorithm, as it excludes occlusive lesions with less than 50% stenosis. Furthermore, thrombotic burden is also not considered in the SYNTAX algorithm, as it only assigns a relatively small score for the presence or absence of thrombus.^8^ Conversely, Gensini score is very representative of total atherosclerotic burden, because it considers lesions as from 25% luminal stenosis,^15,16^ and is significantly associated with average plaque burden and plaque area as measured by intracoronary ultrasound.^17^ High Gensini scores may indicate multivessel disease and an increase in microvascular resistance, both of which are factors associated with NR.^5,10,18^

In the present study, Gensini score was an independent predictor of NR. Modolo et al.^19^ showed that total Gensini score and Gensini score of the culprit artery were higher in individuals with NR than in individuals with optimal reperfusion.^19^ However, the severity of luminal stenosis is not the only angiographic predictor of microvascular dysfunction. In fact, plaque morphological changes such as lipid-rich content, large necrotic core, and large amount of attenuated plaque are also strong predictors of NR,^20,21^ suggesting that altered plaque volume and content cause impaired autoregulation and local release of vasoconstrictors, boosting thrombus formation, microembolization of arterial beds and microvascular obstruction.

In the present study, 58.5% of patients in the NR group had thrombus in the culprit artery; 50.4% of these with large thrombus burden (grades 4 and 5 of the TIMI thrombus grade scale), a possible reason for the association with NR. In a large cohort of patients with STEMI undergoing PCI, large thrombus burden was associated with NR (4.0 vs 0.5, p<0.001) and distal embolization (17.3 vs 3.4, p<0.001).^22^

The SYNTAX score was significantly higher in the NR group than in the optimal reperfusion group (17±10 vs 9±8) and was an independent predictor of NR. In a previous study, the SYNTAX score was a predictor of NR, and a SYNTAX score >21 doubled the risk of developing NR.^9^ Total occlusion of the STEMI-related artery, site of occlusion (left main or left anterior descending coronary artery), presence of thrombus, longer lesions, bifurcation lesions and multivessel disease are factors associated with increased SYNTAX scores and may explain the association with NR.^9,10,23^

In the present study, the NR phenomenon occurred in 32.8% of cases, with TIMI flow <3 or TIMI flow =3 but MBG <2 as angiographic criteria. The incidence of NR is much higher in STEMI than in elective PCI, being reported in 30 to 50% of patients undergoing primary PCI for STEMI.^3^ Rezkalla et al.,^3^ investigating NR in patients with STEMI, found a prevalence of 32% as assessed by TIMI and of 52% by MBG.

Age is an important marker of NR. Older patients have higher plaque burden, diffuse coronary atherosclerosis and severe vascular calcification, which may contribute to microvascular dysfunction.^24,25^ Zhou et al.^6^ identified that age > 65 years (OR= 1.470, 95%CI 1.460-1.490, p=0.007), was an independent predictor of NR.^6^ In our study, in univariate analysis, age predicted NR, but in multivariate analysis this relationship was not maintained.

Delayed reperfusion is associated with NR. Previous studies have shown that patients with longer reperfusion time (> 6 h) show a significant increase in NR.^6,7^ However, a study using a shorter cut-off point (< 6 h) from symptom onset did not indicate delayed presentation as an independent predictor of NR.^23^ In our study, reperfusion time was 2.94 h in the NR group and 2.5 h in the optimal reperfusion group. Multivariate analysis adjusted for GRACE score did not show reperfusion time as a predictor of NR.

The pathophysiology of the NR phenomenon is multifactorial and involves individual susceptibility, ischemia-related injury, reperfusion-related injury, and distal embolization.^26^ During PCI, in the setting of STEMI, distal embolization of thrombus and atherosclerotic plaque components are important mechanisms involved in the pathogenesis of NR.^27,28^ The released atherothrombotic material causes mechanical obstruction, vasoconstriction due to the release of serotonin, thromboxane A2, and endothelin, and endothelial dysfunction due to increased expression of tumor necrosis factor alpha (TNFα).^28-30^ Likewise, the release of platelet- and endothelial-derived microparticles is associated with reduced myocardial perfusion as assessed by MBG and with larger thrombus burden.^31^ The burden of neutrophil extracellular traps may propagate thrombosis and inflammation distally into the culprit artery, contributing to myocyte death.^32,33^

A limitation of this study is that it represents the experience of a single center. Also, coronary angiography has limited ability to estimate both thrombotic and atherosclerotic plaque burden compared with intracoronary ultrasound and optical coherence tomography.

## Conclusion

Atherosclerotic burden assessed by Gensini score and thrombus burden in the culprit artery add predictive value to the SYNTAX score in detecting the NR phenomenon after PCI in patients with STEMI.
